# The Capacity Gain of Orbital Angular Momentum Based Multiple-Input-Multiple-Output System

**DOI:** 10.1038/srep25418

**Published:** 2016-05-05

**Authors:** Zhuofan Zhang, Shilie Zheng, Yiling Chen, Xiaofeng Jin, Hao Chi, Xianmin Zhang

**Affiliations:** 1 College of Information Science and Electronic Engineering, Zhejiang University, Hangzhou, 310027, China

## Abstract

Wireless communication using electromagnetic wave carrying orbital angular momentum (OAM) has attracted increasing interest in recent years, and its potential to increase channel capacity has been explored widely. In this paper, we compare the technique of using uniform linear array consist of circular traveling-wave OAM antennas for multiplexing with the conventional multiple-in-multiple-out (MIMO) communication method, and numerical results show that the OAM based MIMO system can increase channel capacity while communication distance is long enough. An equivalent model is proposed to illustrate that the OAM multiplexing system is equivalent to a conventional MIMO system with a larger element spacing, which means OAM waves could decrease the spatial correlation of MIMO channel. In addition, the effects of some system parameters, such as OAM state interval and element spacing, on the capacity advantage of OAM based MIMO are also investigated. Our results reveal that OAM waves are complementary with MIMO method. OAM waves multiplexing is suitable for long-distance line-of-sight (LoS) communications or communications in open area where the multi-path effect is weak and can be used in massive MIMO systems as well.

In modern society, electromagnetic (EM) waves are well used in many different situations, ranging from fundamental research and development to wireless communications and practical applications. However, there are still properties of the classical EM field that are not fully utilized. We all know that EM waves carry spin angular momentum (SAM) which is connected to the polarization of the electric field^1^,^2^, but not until 1992 when Allen *et al*. recognized that light beams with a azimuthal phase distribution of exp (*ilφ*), where *l* is topological charge and *φ* is the azimuthal angle, carry orbital angular momentum (OAM), did OAM come into our sight^3^. Since then, the techniques using orthogonal OAM states for multiplexing are well studied^4^,^5^,^6^,^7^,^8^,^9^,^10^,^11^. On the physics layer, when OAM is used for information transfer, as long as the receiving aperture is large enough to collect power and phase skewing, it is possible to enhance the channel capacity tremendously within a fixed frequency bandwidth^6^,^12^. Nonetheless, the unavoidable divergence and singularity of OAM-carrying beam will cause receiving problems in a practical situation. In the optical domain, this problem has been overcome with the help of paraxial approximation and the capacity of optical communications is accordingly increased^13^,^14^. Over the last decade, it was found that the photon OAM is not restricted in the optical domain and can be used in radio domain^15^,^16^,^17^. Thidé *et al*. experimentally demonstrated the free space radio communication link using OAM states^18^,^19^,^20^. Theoretically, in an ideal case where the monolithic multi-dimensional antennas at the transmitting and receiving ends can make direct use of the rotation degree of freedom, OAM is a natural unused degree of freedom for wireless communications^12^,^21^. But it is not yet available in a practical situation since an unbearably large aperture of receiving antenna or arrays is required to recover the transmitting signals. Hence there are many scientists arguing about whether OAM radio waves can increase the channel capacity for wireless communications^22^,^23^,^24^,^25^,^26^,^27^. Edfors and Johansson^22^ claim that radio communication over the sub-channels given by OAM states is only a subset of the solutions offered by multiple-in-multiple-out (MIMO) technique. The restrictions of OAM waves multiplexing in radio communications due to crosstalk, disalignment or poor signal-to-noise ratio (SNR) are also studied^23^,^24^. Most recently, Oldoni *et al*. has verified that, with the constraints of the receiver size, an OAM based MIMO radio system is equivalent to conventional MIMO systems in the view of channel spatial multiplexing^28^. As far as we know, no one has proved that a practical OAM utilized multiplexing link has a better performance, in sense of capacity, than the conventional MIMO methods.

However, all these negative arguments are based on the comparison between conventional MIMO system and OAM multiplexing system whose OAM waves are generated by uniform circular array (UCA)^29^,^30^ and transmitted coaxially. In a transmitting UCA or a receiving UCA, every single antenna is fed by a beam-forming network (BFN). This feature leads to the similarity of OAM multiplexing and conventional MIMO^22^,^25^. Besides, the size constrain of UCA compromises the spatial orthogonality of OAM beams^28^. Actually, besides UCA there are many ways to generate OAM waves, for example, spiral phase plate (SPP)^31^,^32^,^33^, helicoidal parabolic antenna^20^,^34^, dielectric resonator^35^, *etc*. In 2014, our group proposed a novel kind of antenna scheme to generate OAM waves^36^,^37^. It is a circular traveling-wave OAM antenna based on ring resonant cavity and able to generate any desirable OAM state but does not need a complex feeding network like UCA does. Accordingly, this kind of antenna increases the opportunity of exploiting the natural properties of OAM waves.

In our study, the circular traveling-wave OAM antennas^37^ are used as the independent elements of transmitting uniform linear array (ULA). Due to the different phase distributions of different OAM states, OAM waves are capable to decrease the mutual correlation of MIMO channel. Therefore, we try to utilize the diversity of OAM waves instead of focusing on its spatial orthogonality^38^. It is found that this kind of OAM based MIMO system can increase communication distance for line-of-sight (LoS) MIMO channel if the OAM states as the elements of the transmitting ULA are sorted in an ordered sequence. In other words, this OAM based MIMO system has a higher capacity gain than the conventional MIMO method under the same system conditions. Although the theoretical maximum capacity limitation of MIMO system cannot be broken, for example, the maximum capacity gain of a 4 × 4 MIMO system is 4 over the single-in-single-out (SISO) channel, the numerical results show that our system has a higher capacity than conventional MIMO system while the communication distance is long enough. By adopting OAM waves, the spatial correlation of LoS channel becomes lower, hence the capacity increases.

As for a conventional MIMO system, a large element spacing (at least half a wavelength) is required to ensure relatively low mutual correlations of sub-channels for LoS channel. This might limit the usefulness of the MIMO technique in practice, especially for the massive MIMO system. On the other hand, the mutual correlation keeps increasing with the propagation distance, and this is because the distances of the sub-channels between each pair of transmitting antenna and receiving antenna are almost the same. Different from the plane waves, OAM waves have a wave vector in the azimuthal direction. Through this particular wave vector, OAM based MIMO system can be seen as a spacing-increased conventional MIMO system. From this point of view, an equivalent model is proposed to explain the causation why the OAM based MIMO system can increase the capacity for LoS MIMO channel. The capacity gains of the OAM based MIMO system and the equivalent spacing-increased conventional MIMO system are compared to verify our model.

In addition, the effects of some system parameters including element spacing and OAM state interval on the performance of the OAM based MIMO system are also studied. The paper is organized as follows, the theoretical background and system configuration of the OAM based MIMO system is first illustrated, then the numerical calculation procedures are presented, and finally the numerical results, equivalent model and discussion of the applications of the proposed system are detailedly demonstrated in proper order.

## Results

### System model

#### Theoretical Background

As described by Maxwell equation, an EM source will radiate angular momentum (AM) as well as linear momentum^39^. The AM of EM field can be observed if its volumetric density carried by the electric and magnetic fields **E**, **B** is integrated over a finite volume^16^, i.e.,


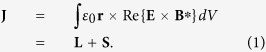


As presented by equation (1), the AM is composed of spin angular momentum **S** and orbital angular momentum **L**,


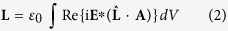



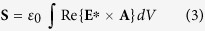


where 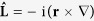 is the OAM operator, and **A** is the vector potential. SAM is intrinsic since it does not depend on the choice of axis, while OAM is extrinsic because its value depends on the choice of calculation axis. By design the OAM transducers (transmitting and receiving antennas) appropriately, OAM can be emitted and sensed in an optimum way. In this paper, the OAM-carrying beam is generated by a circular traveling-wave OAM antenna with a radius *a*^40^. Depending on the azimuthal angle *φ*, the current distribution along the antenna is 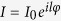, where *I*_0_ is the constant current density. Thus the vector potential of the circular traveling-wave antenna can be written as


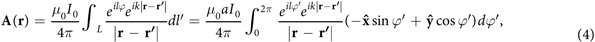


where *μ*_0_ is the magnetic permeability of vacuum. The superscript′ denotes the source coordinate, and point [*r*, *θ*, *φ*] denotes an observation point in spherical coordinate (or [*ρ*, *φ*, *z*] in cylindrical coordinate). Using the standard infinitesimal dipole approximation^16^,^41^, i.e., 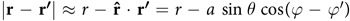 for phases and 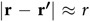 for amplitudes, the vector potential of

equation (4) can be approximated as


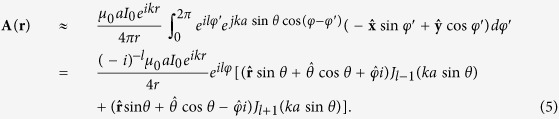


Obviously, 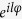 corresponds to the azimuthal phase dependency of the vector potential. If 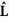 operates on **A**, the azimuthal phase dependence can be observed due to 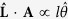. When integrated over the whole beam, the transverse momentums (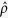 and 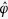 components) vanish because of the rotational symmetry and the only angular momentum is in the direction of propagation (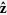 direction). Thus equation (2) becomes 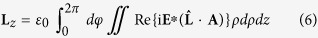


In this way, the approximations of local OAM eigen-modes can be evaluated analytically and the information they carry can be decoded. It should be emphasized that this way of estimating **L**_*z*_ is based on the application of the OAM operator 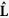 on the electric or magnetic field generated by circular traveling-wave antenna defined by equation (5).

#### System configuration

**Figure 1:**
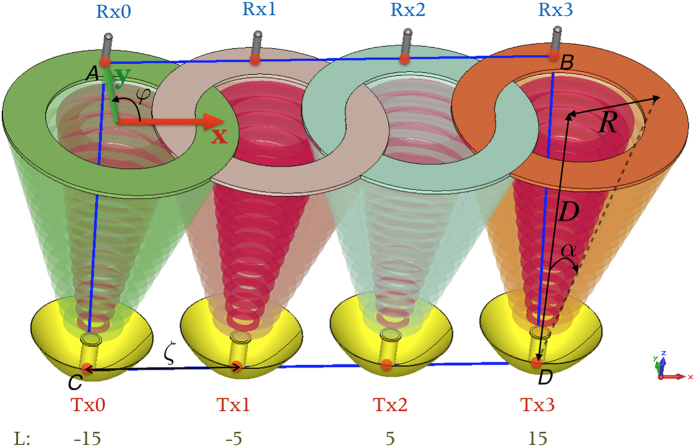
System configuration for the OAM based MIMO system.

An ideal OAM radio communication system (coaxial transmission) does not require digital post-processing, but it needs an impractical large receiving aperture to keep its spatial orthogonality. In order to make full use of the angular phase distribution of OAM but avoid its divergence and singularity problem, an OAM based MIMO system is proposed. Its configuration diagram is depicted in Fig. 1. For simplicity, the diagram is pictured as a 4 × 4 MIMO system. The transmitter is a ULA with *N* OAM antennas, and the receiver is also a ULA but with *N* ordinary MIMO antennas. Every OAM antenna in the transmitting ULA is a circular traveling-wave OAM antenna and able to generate any desired OAM state. The beams generated by the cavities are focused and shaped by a parabolic reflector, by which the spatial divergence angle *α* of different OAM beams can be controlled to be the same (see Fig. 3. of ref. 37 for reference). Since the radiation pattern of the OAM-carrying beams is “doughnut” shaped, given a propagating distance *D*, the middle radius of the doughnut *R*(*D*) is equal to *D* tan*α*. The receiving antennas are placed at the tangency points of line *AB* and the *N* doughnuts’ middle circles of the OAM waves. Note that the element spacings *ζ* of the transmitting ULA and receiving ULA will be the same in such a configuration.

*N* × *N* LoS links are established between the transmitting array and receiving array. Each transmitting antenna is capable of generating a carrier with same frequency but different OAM states. At the receiving end, energy and information from every single transmitting antenna can be collected by each receiving antenna, and MIMO processing is employed to decode the received signals.

Unlike the configuration proposed by Edfors and Johansson^22^, every transmitting antenna in our system is independent, thus we do not need a circular phased antenna array to generate OAM waves and a complex beam-forming network to feed the array. Essentially, it is a MIMO system but replacing normal EM waves with OAM waves. Since the diversity rather than the orthogonality of OAM waves is required in our system, there is no need to consider the crosstalk^24^. As the receiving antennas are placed at where the power density is maximum, signal-to-noise ratio (SNR) is large enough. In other words, such a system is trading orthogonality for power or diversity. For the sake of simplicity, several considerations are assumed on the system:

- The number of antenna elements are the same for both arrays, 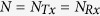.
- Mutual couplings between the transmitting antennas are neglected.
- The radius of the beam is much larger than the element spacing, 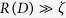. Thus, the differences of link budgets for different sub-channels are neglected.

#### Channel matrix

As for a conventional LoS MIMO system, the channel matrix consists of transfer functions from each transmitting antenna to each receiving antenna^22^. Its system configuration is similar to Fig. 1 but replacing the transmitting OAM antennas with ordinary MIMO antennas. Given the distance *d* between a pair of antenna elements, the transfer function of conventional MIMO system can be expressed as


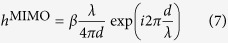


where *β* contains all the variables associated with the antenna system configuration, the free space loss is *λ*/(4*πd*), the exponent stands for the propagation term, and *λ* denotes the wavelength of the carrier wave.

As for the OAM based MIMO system, its transfer function can be derived from the normalization of equation (5)


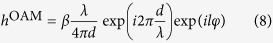


where *l* is the OAM state that can take any integer number, *φ* is the azimuthal angle as shown in Fig. 1 and exp(*ilφ*) corresponds to the phase distribution of the OAM state.

The propagation channel of a *N* × *N* MIMO system can be characterized by a *N* dimensional square channel matrix H. The terms 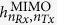 or 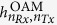 correspond to the propagation from the *n*_*Tx*_-th transmitting antenna to the *n*_*Rx*_-th receiving antenna for the conventional MIMO system and the OAM based MIMO system, respectively. The point-to-point distance and azimuthal angle between the pair of antenna elements is given by


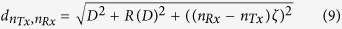



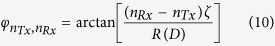



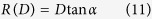


where 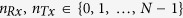, *D* is the relative distance between the two ULAs, *α* is the divergence angle, and *ζ* is the element spacing of the ULA. Thus, the transfer functions of the conventional MIMO channel matrix H^MIMO^ can be denoted as follow by taking equation (9) into equation (7)


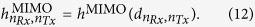


Similarly, the term of the OAM based MIMO system matrix H^OAM^ becomes


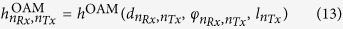


by taking equation (9) and equation (10) into equation (8). And 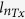 is the OAM state of the *n*_*Tx*_-th transmitting antenna. In the OAM based MIMO system, the antenna array is used for multiplexing rather than generating OAM waves, so the value of 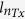 is not restricted by the number of elements in ULA as emphasized in ref. 16 that 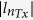 should be smaller than 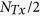. For simplicity, we assume the OAM states of the transmitting antennas are distributed evenly with an OAM state interval Δ*L*. The OAM states vector is denoted as


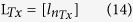


with entries


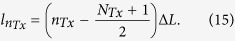


With above matrixes, the performance of the OAM based MIMO system can be compared with that of the conventional MIMO system by calculating their capacities, respectively. For a SISO system, the capacity will be the same whether OAM waves are used or not. So the channel capacities of these two systems, which are operating at the same antenna separation and using the same total transmit power, are compared relative to the capacity of a SISO system. As introduced in ref. 22, this capacity measurement is called the *capacity gain* of the MIMO system over a SISO system.

As a basis for the capacity gain, we assume the SISO system needs a certain transmit power *P*^SISO^ to achieve a certain SNR for different propagating distance *D*. Using the propagation loss as given by equation (7), the required transmit power is denoted as


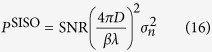


where 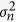 is the receiver noise variance. And the capacity of the reference SISO system is


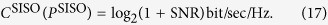


Since the channel matrixes H^MIMO^ and H^OAM^ are already known, the singular value decomposition (SVD)^42^ is used to derive the capacities of both systems. Here we assume the channel state information is known at the transmitter. That is, H is known to both transmitter and receiver. After SVD processing, the positive singular values 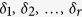 of H are obtained in deceasing order, and 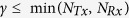 is the rank of H. Therefore, the corresponding capacity for the MIMO system can be described as


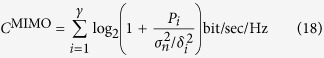


where the total available power 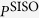 is distributed to the available channels by the water-filling algorithm, such that


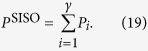


Note that the processing of calculating channel capacity of the OAM based MIMO system is the same as that of MIMO system, but replacing the MIMO channel matrix H^MIMO^ with the OAM waves multiplexing channel matrix H^OAM^ when SVD is processing.

With above equations, the capacities of the conventional MIMO system and the OAM based MIMO system can be evaluated with the same transmit power, i.e., 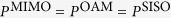. The defined *capacity gain* is given by


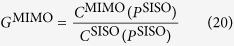



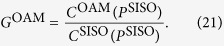


### Comparison of capacity gains

**Figure 2:**
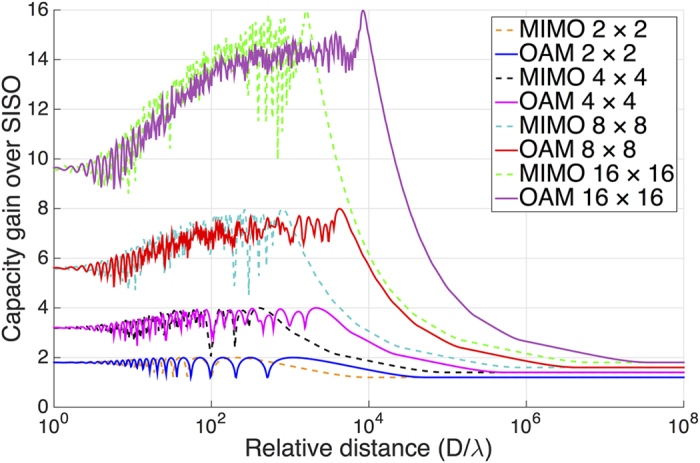
Capacity gains over SISO system for MIMO (dash lines) and OAM based MIMO (solid lines) at ULA sizes 2 × 2, 4 × 4, 8 × 8, and 16 × 16, respectively, at an SNR of 30 dB. Curves are calculated for a state interval Δ*L* = 10, a divergence angle *α* = 2°, an element spacing *ζ* = 10*λ*, and array separation distances from 10^0^ times below to 10^8^ times above the wavelength *λ*.

Based on the definitions of equation (20) and equation (21), Fig. 2 shows the channel capacity gains of conventional MIMO system and OAM based MIMO system for four different configurations with 2 × 2, 4 × 4, 8 × 8, and 16 × 16 antenna elements, respectively. The capacity gains of both systems are calculated at a per-receiver SNR of 30 dB no matter how long the relative distance is.

From the results in Fig. 2, it can be seen that the capacity gains increase with the increasing propagation distance gradually, reach the theoretical maximum, i.e. 2, 4, 8, and 16 times that of a SISO system for the four cases, respectively, and then degrade considerably when the propagation distance exceeds a certain value. The capacity gains of the two compared systems are nearly the same at first, however, the OAM based MIMO system will have a larger capacity gain when the propagation distance is long enough. In other words, to achieve a same capacity gain, the OAM based MIMO system can propagate much longer than the conventional MIMO system under the same system configuration. For example, the OAM based MIMO system can propagate about 5.3 times longer than MIMO system when the capacity gain is 16 times over a SISO system. This result is reasonable as the channel matrix of OAM based MIMO system is more complex (more eigen-modes) owing to the different phase distributions of OAM states. Using OAM waves can decrease the mutual correlation of LoS MIMO channel. Hence, the OAM based MIMO system has a better performance than conventional MIMO system with the evaluation of propagating distance or channel capacity. Besides, it is found that the gaps between the gains of OAM based MIMO and conventional MIMO become larger while the the number of antenna elements of ULA increasing. This result provides a favorable evidence that OAM based MIMO system is very suitable for massive MIMO method since it has a great number of antennas in an array.

The effect of state interval Δ*L* and element spacing *ζ* are also studied under the same system configuration. For convenience, the OAM based MIMO capacity gain over conventional MIMO is defined as


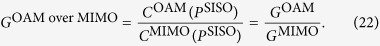


**Figure 3:**
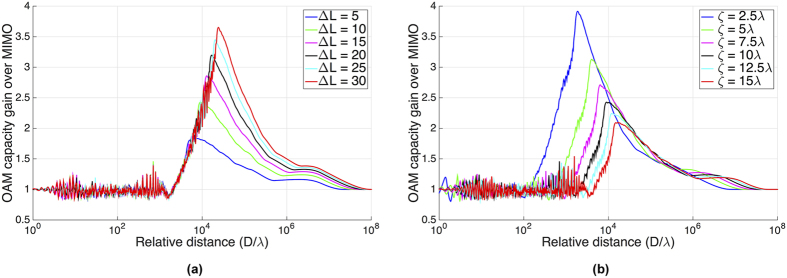
Effects of state interval and element spacing. Curves are calculated for ULA sizes 16 × 16, at an SNR of 30 dB, and a divergence angle *α* = 2°. **(a)** OAM based MIMO capacity gains over conventional MIMO system for different OAM state intervals with an element spacing *ζ* of 10*λ*. **(b)** OAM based MIMO capacity gains over conventional MIMO system for different ULA element spacings with a state interval Δ*L* of 10.

The effect of state interval Δ*L* is shown in Fig. 3(a). As we can see, the larger Δ*L* is, the better the performance of OAM based MIMO system is. That is, the capacity of OAM based MIMO system increases with Δ*L*. It is because the difference of the phase distributions of the transmitting OAM waves becomes bigger and the channel of LoS link becomes more complex when Δ*L* is large. However, there is only one eigen-mode that is useful for communication at a very long distance of 10^8^ times above the wavelength. Thus the OAM based MIMO system has no capacity gain over MIMO no matter how large Δ*L* is, and the only gain available for the channel is the array gain. This effect can be easily illustrated by the equivalent model, which will be demonstrated in the following subsection.

Figure 3(b) gives the effect of element spacing on our system. It can be found that the gain advantage of OAM based MIMO over conventional MIMO becomes more significant when element spacing is small. This is because that the sub-channels between every pair of transmitting antenna and receiving antenna for conventional MIMO system are nearly the same when *ζ* is small. While for the OAM based MIMO system, the difference phase distributions between different OAM beams will still provide the channel independence. However, it is obvious that both OAM based MIMO system and conventional MIMO system cannot propagate a long distance when the element spacing is small. One should balance the tradeoff between capacity gain and propagating distance when designing an OAM waves multiplexing system.

### Equivalent model

**Figure 4:**
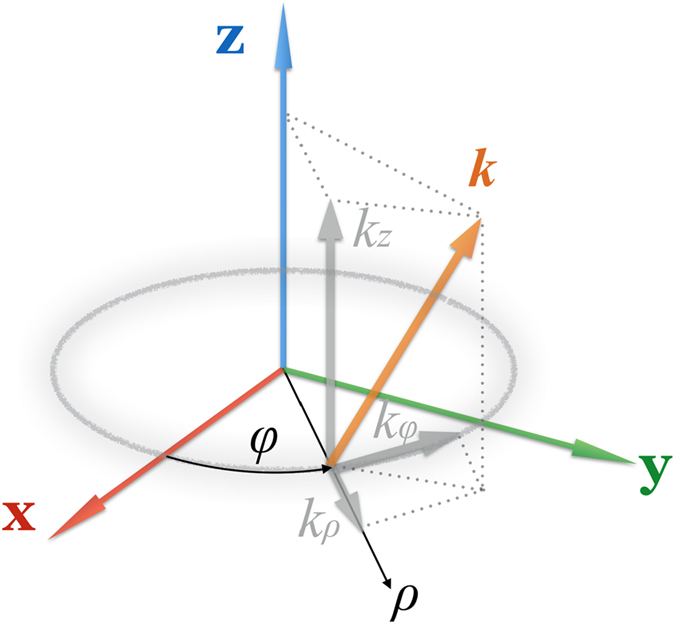
The wave vectors of an OAM beam in cylindrical coordinates.

In order to illustrate the causation why OAM based MIMO system will have a better performance than the conventional MIMO system, an equivalent model is proposed in view of wave vector. As shown in Fig. 4, the wave vector at any point in the cross-section of an OAM beam can be denoted by [*k*_*z*_, *k*_*ρ*_, *k*_*φ*_] in cylindrical coordinate. As for a certain point on the doughnut circle, it can be seen as a infinitesimal element with degree *dφ*. Thus, we can rewrite the exponential index in equation (8) as


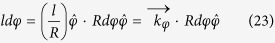


where *R* is the radius, *Rdφ* is the arc length of the infinitesimal element, and 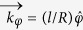 is called the equivalent OAM wave vector.

**Figure 5:**
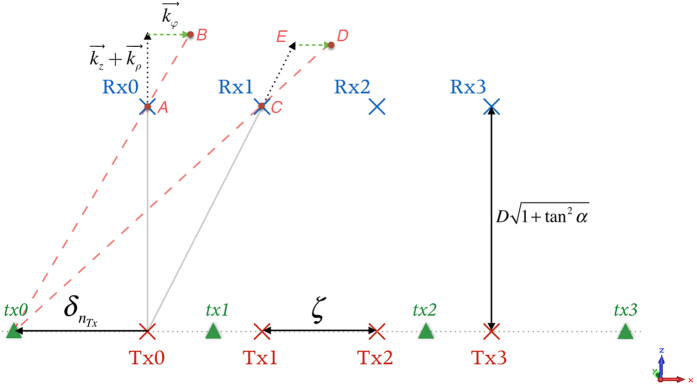
The sketch of the equivalent model. OAM transmitting antennas are denoted as red crosses, and equivalent MIMO antennas are denoted as green triangles.

As shown in Fig. 5, a point-to-point 2D MIMO model can be derived from the 3D model by cutting out plane *ABCD* of Fig. 1. [Tx0, Tx1, Tx2, Tx3] (red crosses) are the transmitting OAM antennas, and [Rx0, Rx1, Rx2, Rx3] (blue crosses) are the receiving MIMO antennas. For any point on the propagating path, 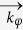 is always vertical to 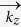 and 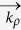. If we lengthen the line segment *AB*, it will intersect *x*-axis at a fixed point *tx0* no matter how long the propagating distance is. As for the sub-channel of transmitting antenna Tx0 to receiving antenna Rx1, the equivalent OAM wave vector 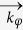 can be denoted as line segment 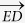 while the radius of the beam *R*(*D*) is much larger than the element spacing *ζ*. Therefore, the intersection point of line *CD* and *x*-axis is still point *tx0*. This can be easily proved by the definition of similar triangles. Hence an OAM antenna placed at Tx0 is equivalent to a conventional MIMO antenna placed at *tx0* by increasing the element spacing of 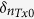. A large OAM state *l* results in a large 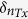, and it is obvious that the equivalent element spacing will increase more while state interval Δ*L* is larger. Similarly, the equivalent MIMO antennas at points *tx1, tx2* and *tx3* can be obtained. As a result, an OAM based MIMO system with transmitting OAM antennas [Tx0, Tx1, Tx2, Tx3] (red crosses) is equivalent to a conventional MIMO system with ordinary antennas [*tx0, tx1, tx2, tx3*] (green triangles). That is to say, the OAM based MIMO system is equal to a conventional MIMO system with a larger element spacing. As for a conventional MIMO system, the larger the element spacing is, the lower the spatial correlation is. Therefore, the mutual correlation will decrease while OAM waves are used in a MIMO system.

**Figure 6:**
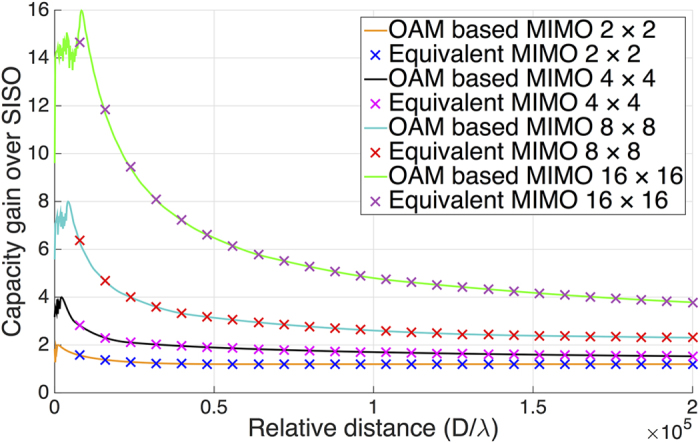
Verification of the OAM equivalent MIMO model (crosses points) at an element spacing *ζ* of 10*λ*, a state interval Δ*L* of 10, an SNR of 30 dB, and a divergence angle *α* = 2°. This picture is plotted with linear axes for convenience.

To verify the correctness of the equivalent model, the capacities of OAM based MIMO system and its equivalent MIMO system are compared as shown in Fig. 6. For convenience, this figure is plotted with linear axes. The OAM based MIMO system is denoted by the solid line, and the equivalent spacing-increased MIMO system is denoted by crosses. Obviously, the capacity of the OAM based MIMO system and that of its equivalent model coincide very well when propagating distance *D* is much longer than element spacing *ζ*.

**Figure 7:**
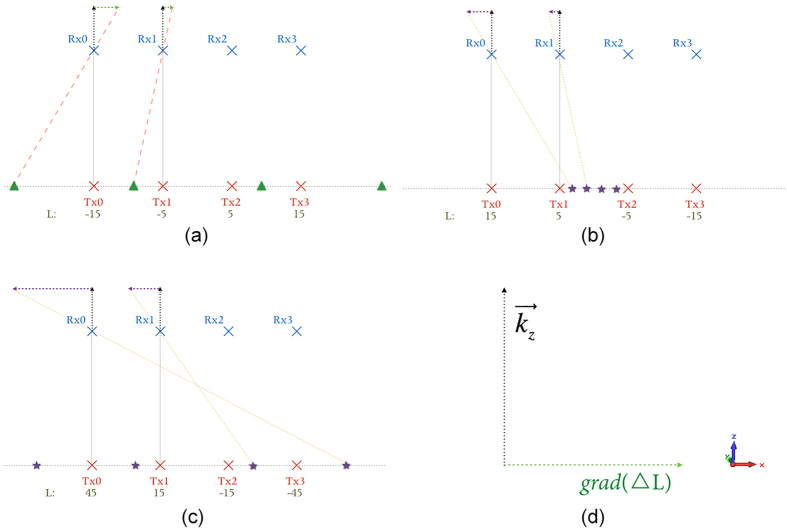
Equivalent models for different configurations. OAM transmitting antennas are denoted as red crosses, and equivalent MIMO antennas are denoted as green triangles or purple stars. **(a)** Δ*L* = 10, *grad*(L_*Tx*_) is the positive direction of *x*-axis. **(b)** Δ*L* = 10, *grad*(L_*Tx*_) is the negative direction of *x*-axis. **(c)** Δ*L* = 30, *grad*(L_*Tx*_) is the negative direction of *x*-axis. **(d)** Configuration of the Lemma.

The OAM states vector L_*Tx*_ of the system in Fig. 5 is [−15, 5, 5, 15] as shown in Fig. 7(a). But if we sort the L_*Tx*_ in decreasing order [15, 5, −5, −15], as shown in Fig. 7(b), the element spacing of the equivalent model will decrease and the capacity gain will decrease accordingly. We may increase the state interval Δ*L* to make the equivalent element spacing increase in the reverse direction like Fig. 7(c) shows, but it does not make full use of the OAM degree of freedom. Therefore, we denote the increasing direction of states vector, positive direction of *x*-axis in Fig. 7(a) or negative direction of *x*-axis in Fig. 7(b,c), as *grad*(L_*Tx*_), and propose a lemma to arrange the direction of *grad*(L_*Tx*_) and the propagating direction 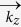, which is shown in Fig. 7(d).

**Lemma.** *To take full advantage of* 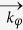
*, the direction of the product of grad*(L_*Tx*_) *and* 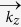 *should be the same as the negative direction of y-axis*.

In addition, by comparing Fig. 7(b,c), we can find that large Δ*L* will lead to large spacing increment 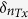. And large 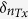 will lead to an equivalent MIMO system with large element spacing. This result is consistent with the analysis about the effect of state interval Δ*L* which we discussed in the last subsection.

## Discussion

The performance of the OAM waves based MIMO system, especially the capacity gain, are thoroughly studied in this paper. In most OAM used communication links, the orthogonality of OAM states is used to encode many channels on the same frequency. Signals are modulated in different orbital angular momentum states and simultaneously transmitted in independent radio channels coaxially. At the receiving end, OAM waves are demodulated by OAM antennas or interferometric phase discrimination method. However, it is widely accepted that the orthogonality of such an OAM system is restricted by the size of receiver and the UCA based OAM multiplexing is equal to a conventional MIMO system thus has no capacity advantage than MIMO. The choice between OAM and conventional MIMO is only a matter of signal processing complexity. Actually, OAM waves are much more diverse than normal EM waves on account of its azimuth phase dependency of exp(*ilφ*). The spatial correlation of the sub-channels will become lower if the discrepant OAM waves are used instead of normal EM waves in a MIMO system. By investigating the performance of an OAM based MIMO system in comparison with a conventional MIMO system, it is found that OAM based MIMO system has a higher capacity gain and can propagate longer under the same conditions. An equivalent model is also proposed to verify the capability of OAM waves multiplexing. OAM waves have an equivalent OAM wave vector 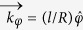, which is vertical to the general wave vector components 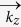 and 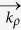. With this special wave vector, the OAM based MIMO system is equivalent to an spacing-increased conventional MIMO system, or, conversely, OAM based MIMO system needs a smaller spacing to achieve a given capacity gain.

All results in this paper are based on the assumption that LoS channel from transmitter to receiver is totally free from reflection. What happens if reflections are present remains to be further investigated but we believe reflection could enhance the spectral density of OAM radio communications and the capacity gain would be higher in consideration of the mirror image theory^43^. Another fundamental assumption of the OAM based MIMO system is that every OAM antenna in the transmitting array is independent and capable to generate pure OAM waves without relying on other antennas. For simplicity, we only study the ULA structure in this paper, which is a 1D MIMO setting. However, in our opinion, other array structures of OAM multiplexing are also capable to increase the channel capacity as long as their elements are independent and satisfy the lemma we discussed above.

To summarize, we may reach the conclusion in this paper that the OAM based MIMO system has a higher capacity gain than the conventional MIMO method if we utilize the diversity of OAM waves but not the orthogonality. And OAM waves multiplexing is complementary with the conventional MIMO system. Since the capacity gain advantage of OAM based MIMO system over conventional MIMO system becomes more significant while the number of antenna elements increasing or the element spacing decreasing, we believe OAM based MIMO system will have a great potential in massive MIMO method which requires a great number of antennas and limited spatial spacings between antennas. OAM based MIMO is also very suitable for the communications in open area or long-distance communications where the multi-path effect is weak and conventional MIMO does not work anymore. Since small element spacing and large state interval will lead to a better performance of OAM based MIMO system, we think one of the significant tasks for OAM based radio communications in the future is to design an OAM antenna which can generate large OAM state but has a tiny size.

## Methods

The OAM waves are generated by the circular slot antenna, which is designed by CST Microwave Studio. All numerical results are calculated by MATLAB.

## Additional Information

**How to cite this article**: Zhang, Z. *et al*. The Capacity Gain of Orbital Angular Momentum Based Multiple-Input-Multiple-Output System. *Sci. Rep*. **6**, 25418; doi: 10.1038/srep25418 (2016).
